# Cross-sectional study of prevalence and determinants of uncontrolled hypertension among South African adult residents of Mkhondo municipality

**DOI:** 10.1186/s12889-020-09174-7

**Published:** 2020-07-06

**Authors:** Charity Masilela, Brendon Pearce, Joven Jebio Ongole, Oladele Vincent Adeniyi, Mongi Benjeddou

**Affiliations:** 1 Department of Biotechnology, University of the Western Cape, 7535, Bellville, Cape Town, South Africa; 2 Department of Family Medicine, Center for Teaching and Learning, Piet Retief Hospital, Mkhondo, South Africa; 3 Department of Family Medicine, Walter Sisulu University, East London, South Africa

**Keywords:** Blood pressure control, Dyslipidaemia, Mpumalanga, South Africa, Uncontrolled hypertension

## Abstract

**Background:**

Achieving the blood pressure treatment target in individuals with hypertension is a serious global health challenge. Furthermore, the actual burden of uncontrolled hypertension is poorly understood, especially in the developing countries. Therefore, this study comprehensively examined the prevalence and factors associated with uncontrolled hypertension in individuals receiving care at the primary healthcare facilities in the rural areas of Mkhondo Municipality in the Mpumalanga Province, South Africa.

**Methods:**

In this cross-sectional study, 329 individuals attending care for hypertension were recruited from January 2019 to June 2019 at three primary healthcare centres, namely, Piet Retief hospital, Mkhondo town clinic and Thandukukhanya community health centre. Uncontrolled hypertension was defined as systolic blood pressure ≥ 140 mmHg and/or diastolic blood pressure ≥ 90 mmHg in accordance with the South African Hypertension Society guideline (2014). Multiple logistic regression (Forward LR method) analysis was used to identify the significant determinants of uncontrolled hypertension.

**Results:**

The majority of the participants were 55 years old and above (69.0%), Zulus (81.2%), non-smokers (84.19%) and had been diagnosed with hypertension for more than a year prior to the study (72.64%). The overall prevalence of uncontrolled hypertension was 56.83% (*n* = 187) with no significant difference between sexes, 57.38% male versus 56.88% female, respectively. In the multiple logistic regression model analysis after adjusting for confounding variables, obesity (AOR = 2.90; 95% CI 1.66–5.05), physical activity (AOR = 4.79; 95% CI 2.15–10.65) and HDL-C (AOR = 5.66; 95% CI 3.33–9.60) were the significant and independent determinants of uncontrolled hypertension in the cohort.

**Conclusion:**

The high prevalence of uncontrolled hypertension in the study setting can be largely attributed to obesity, physical activity and dyslipidaemia. Treatment will require the collaborative efforts of individuals, clinicians and health authorities. All these determinants should be addressed decisively so as to achieve the treatment blood pressure targets in the study population.

## Background

The prevalence of non-communicable diseases (NCDs) is increasing at an alarming rate world-wide and it has been driven by the rise in the incidence of cardiovascular risk factors such as obesity, diabetes and hypertension [1]. Hypertension is one of the important risk factors for morbidity and mortality, affecting about 1 billion people world-wide [2]. In the year 2000, 972 million people were living with hypertension and a large burden was borne by economically disadvantaged countries [2], where awareness and treatment often fall short. According to the World Health Organization (WHO), 27.4% of men and 26.1% of women have hypertension [3], although an overall prevalence of up to 54% has been reported in urban areas [4]. In the absence of effective intervention strategies to control this epidemic, the prevalence of hypertension is likely to increase in the next decade [2, 4].

Hypertension is a multifactorial and highly complex disease that is characterized by a systolic blood pressure ≥ 140 mmHg and/ or diastolic blood pressure ≥ 90 mmHg [5]. The risk factors of hypertension include excessive salt intake [6], alcohol consumption and lack of physical activity [7]. Evidence suggests that hypertension has no obvious symptoms and often remains undiagnosed for a long period [8]. Furthermore, the asymptomatic and persistent nature of the disease presents a major challenge of identifying people with elevated blood pressure and providing optimal medical care [8]. When diagnosed early, lifestyle changes and pharmacological interventions are essential for the management and control of the disease [8, 9]. However, poor adherence to non-pharmacological and pharmacological management of hypertension represents a serious challenge for public health in many countries [10, 11].

Uncontrolled hypertension, defined as blood systolic blood pressure ≥ 140 and / or diastolic blood pressure ≥ 90 mmHg, has been associated with patients’ aging, clinical inertia, unemployed status and nutritional transitions [12]. Furthermore, only 25% of individuals undergoing anti-hypertensive treatment appear to be well controlled [9], while the majority of studies conducted in Africa have shown that less than a third of patients reach their treatment goals [13]. In South Africa, the prevalence of uncontrolled hypertension has been estimated at between 13.5–75.5% [12, 14], whilst figures ranging between 19.0–56.0% have been reported for hypertension control [3]. On the other hand, hypertension control among individuals residing in high-income countries has been reported as being as high as 82.0% [3, 15], whereas a number as low as 28.4% has also been reported in high-income countries [16]. Those who fail to reach therapeutic targets continue to be at higher risk of cardiovascular events, kidney diseases [9, 17], stroke [18], metabolic syndrome, hypertensive retinopathy [19] and dementia [20]. In addition to adherence, the multifactorial nature of hypertension presents the greatest challenge for its treatment and control [21]. Even so, only a few studies have assessed the prevalence of uncontrolled hypertension and its determining factors in South Africa, particularly within economically disadvantaged populations. As a result, the actual burden of uncontrolled hypertension in such communities is poorly understood and, awareness as well as treatment to achieve control, remains suboptimal. Therefore, epidemiological data from these communities are essential in planning interventions based on local influential factors associated with the disease. On these grounds, the current study aims to comprehensively assess the prevalence, the socio-demographic and clinical determinants of uncontrolled hypertension among patients receiving health care in three public sector facilities serving the rural areas of Mkhondo municipality in the Mpumalanga province, South Africa.

## Methods

### Study design and settings

This was a community based cross-sectional study conducted in the Piet Retief Hospital, Mkhondo town clinic and Thandukukhanya Community Health Center in Mkhondo Municipality of Mpumalanga Province, South Africa, from January 2019 to June 2019. These health care facilities provide chronic care services for the residents of Mkhondo Municipality. Mkhondo is located in the Gert Sibande district of the Mpumalanga province and is a resource-constrained community made up of a small town surrounded by farms and two townships (Ethandukukhanya and AJAX) with a population of approximately 189,036 [22].

### Study population and sample size estimation

The study included individuals who were aged ≥18 years, had been diagnosed and initiated on treatment for hypertension at least a year prior to the study. Individuals who were bedridden, mentally compromised, pregnant or unable to give consent were excluded from the study.

A sample size of 334 was estimated by using the formula for cross-sectional study:

$$ \left\{\mathbf{N}={\left({\mathrm{Z}}_{1-}\boldsymbol{\upalpha} \right)}^{\mathbf{2}}\mathbf{x}\ \mathbf{P}\ \left(\mathbf{1}-\mathbf{P}\right)/{\mathbf{D}}^{\mathbf{2}}\right\} $$

Given that the prevalence of uncontrolled hypertension ranged widely from 13.5–75.5% in South Africa [12, 14], P (*P* = proportion with uncontrolled hypertension) of 68% was chosen for the sample size estimation. Z_1 –_ α = 1.96 and D = absolute precision, which is taken as 0.05. Five participants dropped out of the study so a total of 329 patients were included in the final analysis.

### Data collection

Eligible participants were recruited sequentially at the study settings over the study period. Trained research assistants conducted face-to-face interviews with consenting participants by using a standardized questionnaire (Supplementary file). The questionnaire comprises socio-demographic characteristics of age, sex, level of education and employment status, lifestyle behaviors; physical activity and dietary patterns; cigarette smoking status and alcohol use; plus, questions about family history of hypertension. The following clinical data were obtained from the medical records: duration of hypertension, number of anti-hypertensive drugs and drug combinations. Drug combinations included the following: Thiazide diuretic only, combination therapy (Thiazide + calcium channel blocker + Angiotensin converting enzyme inhibitor and Thiazide + calcium channel blocker + Angiotensin converting enzyme inhibitor + beta-blocker) and other (Loop diuretic monotherapy, loop diuretic + calcium channel blocker and/ or beta-blocker + calcium channel blocker + Angiotensin converting enzyme inhibitor).

The level of education was categorized as no education, primary (grade 1–6), secondary (7–12) and tertiary. Employment status was categorized as unemployed, employed or receiving social grants from the government. Smoking status were categorized as never smoked or ever smoked; while alcohol use was also categorized similarly. Physical activity was categorized as active (if engaging in vigorous intensity exercise leading to an increase in heart and respiratory rate, such as gardening, or inactive (not engaging in any physical activity). Also, participants reported their average consumption of fruits and vegetables, as well as fast food and salt intake.

A trained research nurse conducted anthropometric measurements of weight to the nearest 0.1 kg using a digital scale (Tanita-HD 309, Creative Health Products, MI, USA) and height to the nearest of 0.1 cm using a mounted stadiometer. All measurements were taken with the participants wearing minimal clothing and no shoes. Body Mass Index (BMI) for each patient was calculated as weight (kg) divided by height in meters squared (m^2^) and was categorized based on WHO criteria as obese (30 or greater kg/ m^2^) or not [23].

Blood pressure (BP) was measured using a validated automated digital blood pressure monitor (Macrolife BP A 100 Plus model) according to standard protocols. The BP was recorded in triplicate and the average was used for analysis. Patients with systolic BP (SBP) of ≥140 mmHg and/ or diastolic BP (DBP) ≥ 90 mmHg were defined as uncontrolled BP [24].

### Laboratory assessment

Patients participated in an 8 h fast before the research nurse drew five millilitres of venous blood from each of them as a sample for laboratory assessment. The lipid profile, which includes total cholesterol (TC), triglycerides (TG), low-density lipoprotein (LDL-C) and high-density lipoprotein (HDL-C)] for each participant, was categorized according to the guidelines of The Society for Endocrinology, Metabolism and Diabetes of South Africa (SEMDSA, 2017). All laboratory assays were conducted by the National Health Laboratory Services (NHLS) of Piet Retief and Ermelo Provincial hospitals in accordance with standard protocols.

### Statistical analysis

Data analysis was conducted using IBM SPSS Statistics for Windows, Version 25.0 (IBM Corp., Armonk, New York, USA). General characteristics of the participants were expressed as mean ± standard deviation for continuous variables. Categorical variables were reported as frequency (percentage). The associations between socio-demographic-, clinical factors and uncontrolled hypertension were examined by using bivariate analysis. Multiple logistic regression odds ratios and their 95% confidence intervals, using crude and adjusted logistic regression model analysis, helped identify the independent determinants of uncontrolled hypertension. A *p*-value of less than 0.05 was considered statistically significant.

## Results

Of the total number of participants (*n* = 329), 61 were males (18.5%) and 268 were females (81.5%). The majority of the participants were aged 55 years and above (69.0%), of Zulus origin (81.2%), non-smokers (84.19%), non-alcohol drinkers (77.81%), consuming fruit and vegetables (97.87%) and fast food about three times per week (61.09%), which differed according to sex. A sedentary lifestyle was reported by 231 participants (70.21%); 22 were males and 209 were females. Consumption of excessive amounts of salt was reported by 42 participants (12.78%) of whom a higher proportion were women (*n* = 37) in comparison with men (*n* = 5). The majority of the participants who had been diagnosed within the previous 5 years (*n* = 234; 72.64%) were predominantly women (*n* = 194). Table 1 provides detailed descriptive characteristics of the participants.

**Table 1:** Demographic characteristics of the study participants disaggregated by hypertension control

Variables	All Participants (n; %)	Controlled Hypertension (n; %)	Uncontrolled Hypertension (n; %)	***p***-value
All	329 (100%)	142 (43.16%)	187 (56.83%)	
Gender				0.925
Male	61 (18.54)	26 (18.31)	35 (18.72)	
Female	268 (81.46)	116 (81.69)	152 (81.28)	
Age (Years)				0.467
18–25	08 (2.43)	03 (2.11)	05 (2.67)	
26–35	13 (3.95)	05 (3.52)	08 (4.28)	
36–45	29 (8.81)	08 (5.63)	21 (11.22)	
46–55	69 (20.97)	32 (22.54)	37 (19.78)	
56–65	97 (29.48)	47 (33.09)	50 (26.73)	
≥ 66	113 (34.35)	47 (33.09)	66 (35.29)	
Ethnicity				0.813
Zulu	267 (81.16)	114 (80.28)	153 (81.81)	
Swati	54 (16.41)	24 (16.90)	30 (16.04)	
Not specified	08 (2.43)	4 (2.82)	4 (2.14)	
Employment status				0.201
Employed	79 (24.01)	39 (27.46)	40 (21.39)	
Unemployed	104 (31.61)	38 (26.76)	66 (35.29)	
Social grant recipient	146 (44.38)	65 (45.80)	81 (43.32)	
Educational Level				0.157
Tertiary	07 (2.13)	05 (3.52)	02 (1.07)	
Secondary	109 (33.13)	46 (32.39)	63 (33.68)	
Primary	140 (42.55)	54 (38.03)	86 (45.98)	
Illiterate	73 (22.19)	37 (26.05)	36 (19.26)	
Smoking status				0.634
Never Smoked	277 (84.19)	118 (83.09)	159 (85.03)	
Ever Smoked	52 (15.81)	24 (16.91)	28 (14.97)	
Alcohol consumption				0.922
Never Drank	254 (77.20)	110 (77.46)	144 (77.01)	
Occasional	75 (22.80)	32 (22.54)	43 (22.99)	
Fruit and Vegetable Consumption				0.987
1–3 times/week	322 (97.87)	139 (97.89)	183 (97.86)	
Never	07 (2.13)	03 (2.11)	04 (2.14)	
Fast Food Consumption				0.459
Never	128 (38.91)	52 (36.62)	76 (40.64)	
1–3 times/week	201 (61.09)	90 (63.38)	111 (59.36)	
Salt intake				0.771
Low-Moderate	287 (87.23)	123 (86.62)	164 (87.70)	
Increased	42 (12.77)	19 (13.38)	23 (12.30)	
Duration of Diagnosis				0.140
< 5 years	95 (28.88)	35 (24.65)	60 (32.09)	
≥ 5 years	234 (71.12)	107 (75.35)	127 (67.91)	

### Prevalence of uncontrolled hypertension

**Fig. 1:**
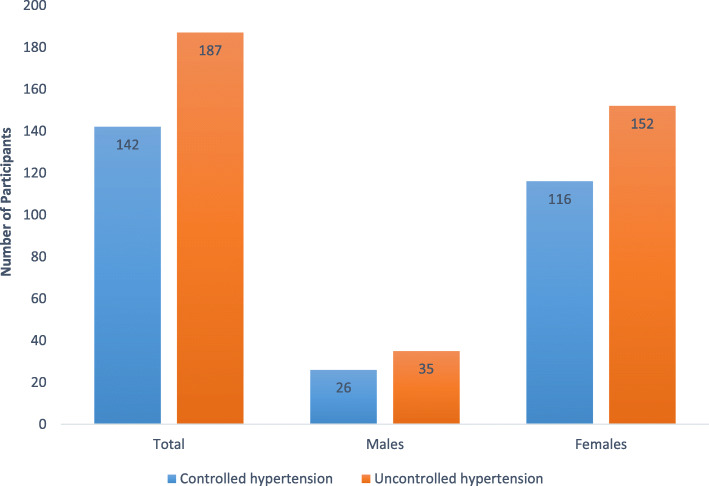
Prevalence of Uncontrolled hypertension

All the participants (*N* = 329) had been on anti-hypertensive treatment for at least a year. The majority of the participants were on at least two or more anti-hypertensive drugs (*n* = 260; 79.03%). Thiazide diuretic (for example, hydrochlorothiazide) was the preferred drug class, either alone (*n* = 44; 13.37%) or in combination with other drugs (calcium channel blockers, beta-blockers and Angiotensin-converting enzyme inhibitors) (86.62%). Successful treatment of reaching the blood pressure target of 140/90 mmHg occurred in 142 patients (43.62%) of the 329 participants. The overall prevalence of uncontrolled hypertension was 56.83% (*n* = 187), with no significant difference between sexes: 57.38% males versus 56.88% females, respectively (Fig. 1).

### Factors associated with uncontrolled hypertension

In the bivariate logistic regression analysis (Table 2), physical activity (*p* < 0.001), positive family history (*p* = 0.034), low HDL-C (*p* < 0.001) and obesity (*p* = 0.001) were significantly associated with uncontrolled hypertension. Other risk factors such as age, gender, smoking status, alcohol consumption, Total cholesterol, LDL-C and triglycerides were not significantly associated with uncontrolled hypertension (*p* > 0.05).

**Table 2:** Bivariate analysis showing the factors associated with uncontrolled hypertension

Variables	Controlled Hypertension (n; %)	Uncontrolled Hypertension (n; %)	95%CI	***p***-value
Family history				
Negative	90 (63.38)	132 (70.59)	1	
Positive	52 (36.62)	55 (29.41)	1.91 (1.04–3.49)	**0.034**
Total Cholesterol				
< 5.2 mmol/L	101 (71,13)	131 (70.05)	1	
≥ 5.2 mmol/L	41 (28.87)	56 (29.94)	1.12 (0.53–2.37)	0.759
HDL-C				
≥ 1 mmol/L	100 (70.42)	59 (31.55)	1	
< 1 mmol/L	42 (29.58)	128 (68.45)	0.13 (0.07–0.24)	**< 0.001**
LDL-C				
< 2.6 mmol/L	69 (48.59)	86 (45.99)	1	
≥ 2.6 mmol/L	73 (51.41)	101 (54.01)	0.83 (0.42–1.64)	0.596
Triglycerides				
< 1.7 mmol/L	65 (45.77)	74 (39.57)	1	
≥ 1.8 mm0/L	77 (54.23)	113 (60.43)	0.74 (0.40–1.37)	0.342
Drug combinations				
Thiazide	23 (16.20)	21 (11.23)	1	
Thiazide+CCB + ACEI	23 (16.19)	47 (25.13)	1.37 (0.40–4.72)	0.610
Thiazide+CCB + ACEI+β-blocker	04 (2.82)	14 (7.49)	0.65 (0.20–2.14)	0.485
Other	92 (64.79)	105 (56.15)	5.09 (0.15–173.39)	0.366
Number of Drugs				
1	38 (26.76)	31 (16.58)	1	
2	69 (48.59)	68 (36.36)	1.13 (0.04–32.33)	0.943
3	30 (21.13)	73 (39.04)	1.55 (0.06–39.10)	0.788
4	05 (3.52)	15 (8.02)	6.33 (0.22–181.92)	0.281
Diabetes				
No	80 (56.34)	116 (62.03)	1	
Yes	62 (43.66)	71 (37.97)	1.18 (0.65–2.14)	0.587
Obesity				
No	66 (46.48)	56 (29.95)	1	
Yes	76 (53.52)	131 (70.05)	0.35 (0.19–0.66)	**0.001**
Physical Activity				
Inactive	121 (85.21)	127 (67.91)	1	
Active	21 (14,79)	60 (32.09)	0.18 (0.08–0.43)	**< 0.001**

*HDL-C* High density lipoprotein cholesterol; *LDL-C* Low density lipoprotein cholesterol

In the multiple logistic (crude and adjusted) regression model analysis (Table 3), the categories were merged to create a binary outcome for each of the variables, namely, education, employment and age. In the final model, obesity (1.29–3.20), physical activity (1.56–4.75), low HDL-C (3.21–8.30), combination regimen (Thiazide, calcium channel blockers and Angiotensin converting enzyme inhibitors) (1.01–3.17) and being on two (0.09–0.83) or three drugs (0.11–0.95) were the independent and significant determinants of uncontrolled hypertension. However, after adjusting for confounding factors (level of education, ethnicity, smoking status, alcohol use, fruit and vegetable consumption, family history of hypertension, total cholesterol, triglycerides and LDL-C), obesity (1.66–5.05), physical activity (2.15–10.65) and low HDL-C (3.33–9.60) were the significant and independent determinants of uncontrolled hypertension in the cohort. Individuals who were obese were three times more likely to have uncontrolled hypertension compared to those who were not. Likewise, participants who were physically active were close to five times more likely to have uncontrolled hypertension compared to those who were physically inactive. Individuals with low HDL-C were close to six times more likely to have uncontrolled hypertension compared to those with normal HDL-C.

**Table 3:** Adjusted and unadjusted logistic regression models showing the factors associated with uncontrolled hypertension

Variables	Unadjusted odds ratios (95% CI)	Adjusted odds ratios (95% CI)
All		
Gender		
Male	1.03 (0.59–1.80)	1.00 (0.51–1.94)
Female	1	1
Age		
< 55	0.94 (0.59–1.51)	0.85 (0.48–1.48)
≥ 55	1	1
Employment status		
Unemployed	1.49 (0.93–2.41)	1.47 (0.83–2.62)
Employed	1	1
Duration since diagnosis		
> 5 years	0.69 (0.42–1.13)	1.99 (0.94–4.20)
5 years and below	1	1
Salt intake		
Increased	0.91 (0.47–1.74)	1.64 (0.72–3.71)
Low-Moderate	1	1
Diabetes		
Yes	0.79 (0.51–1.23)	0.86 (0.50–1.48)
No	1	1
Obesity		
Yes	2.03 (1.29–3.20)*	2.90 (1.66–5.05)***
No		
Physical Activity		
Active	2.72 (1.56–4.75)***	4.79 (2.15–10.65)***
Inactive	1	1
HDL-C		
< 1 mmol/L	5.17 (3.21–8.30)***	5.66 (3.33–9.60)***
≥ 1 mmol/L	1	1
Drug Combinations		
Thiazide	0.80 (0.42–1.54)	1.32 (0.42–4.12)
Thiazide+CCB + ACEI	1.79 (1.01–3.17)*	0.67 (0.23–1.92)
Thiazide+CCB + ACEI+ β-Blocker	3.07 (0.98–9.64)	2.00 (0.07–57.94)
Other	1	1
Number of Drugs		
1	0.27 (0.09–0.83)*	0.38 (0.02–9.92)
2	0.33 (0.11–0.95)*	0.54 (0.02–12.44)
3	0.81 (0.27–2.43)	1.71 (0.07–43.64)
4	1	1

***p-values < 0.001; *p-values< 0.05; *CI* Confidence Interval; *HDL-C* High density lipoprotein cholesterol; *LDL-C* Low density lipoprotein cholesterol

## Discussion

Hypertension is an independent risk factor for cardiovascular diseases and all causes of premature deaths [2, 7, 18]. As such, the control of hypertension is essential in lowering cardiovascular and mortality risk in patients [18, 21]. However, the prevalence and factors that determine uncontrolled hypertension in rural communities of South Africa are understudied and the actual burden of the disease is poorly understood. This study therefore aimed to assess the prevalence, socio-demographic- and clinical determinants of uncontrolled hypertension among patients receiving healthcare in three government facilities serving the rural areas of Mkhondo municipality in the Mpumalanga province, South Africa.

South Africa has the highest prevalence of hypertension in Southern Africa [4]. Despite the high prevalence, the level of awareness and control of the disease is low among impoverished communities [3]. In the current study, the overall prevalence of uncontrolled hypertension was 56.83%. In comparison to other studies conducted in Africa, the figure presented in this study was slightly lower than those reported in Zimbabwe (61.0%) [25] and Nigeria (60.0%) [26], but higher than the 52.7% reported in Ethiopia [27]. In comparison to other studies conducted in different parts of South Africa, the result is lower than the 75.5% that was reported in the rural Eastern Cape province [12]; however, it is higher than the 13.5% prevalence that was reported in the South African National Health and Nutrition Examination Survey [14]. It is important to note that the wide disparity observed between the two results can be explained by the large sample of non-hypertensive individuals in the national survey, whilst the current study was exclusive to hypertensive patients.

In the current study, the presence of obesity was significantly associated with uncontrolled hypertension. These findings are supported by studies conducted in Ethiopia [13, 27], China [28] and Zimbabwe [29], where excessive body weight was statistically associated with the incidence of uncontrolled hypertension [28, 29]. Moreover, it has been shown that a modest weight-loss will not only decrease blood pressure, but will also have a favorable impact on obesity related risk factors [29, 30]. Obesity is traditionally defined as the abnormal accumulation of body fat ≥20% over the individual’s ideal body weight and assessed as BMI ≥ 30 kg/m^2^ [29]. The increasing prevalence of obesity has been identified as the most important risk factor for a number of life threatening conditions, including hypertension [31, 32]. Patients with obese-related hypertension often present with increased blood volume, high levels of circulating aldosterone, insulin resistance and obstructive sleep apnea syndrome [33, 34]. A number of studies have identified that there is a direct and apparent dose-response relationship between an increase in BMI and blood pressure [35].

In the current study, engagement in physical activity was associated with the higher odds of having uncontrolled hypertension. It should be noted that the activity level of the participants was obtained by self-report and there was no standardized measure of the intensity of the physical activity. In addition, there is a lack of formal recreational centres (gymnasium) for graded exercises in the study setting. Also, other risk factors, such as excessive salt intake, may have masked the beneficial effect of exercise on blood pressure control in this population. A study conducted in Ethiopia showed that non-adherence to physical activity was associated with uncontrolled hypertension [13]. In addition, a study conducted in China showed that lack of physical activity was significantly associated with uncontrolled hypertension [28]. The exact mechanism through which physical activity alleviates high blood pressure has not yet been fully elucidated. In addition, optimal prescription of physical activity for hypertension control is not known. Current hypotheses suggest that there is a link between hypertension control and alterations in insulin sensitivity, autonomic nervous system function and vasoconstriction regulation [13]. There seems to be more evidence supporting the beneficial effects of physical activity on hypertension prevention than those refuting it. Engagement in physical activity for at least 30–45 min, five times per week is associated with a lower incidence of hypertension [36]. In the last few decades, there has been a great deal of evidence on the protective effect of physical activity on the development of hypertension [36, 37]. As a result, physical activity has become the most common lifestyle modification that is often recommended after an individual has been diagnosed with hypertension [37].

Furthermore, the current study found an association between decreased HDL-C (< 1 mmol/L) (dyslipidaemia) and uncontrolled hypertension. Dyslipidemia comprises having an abnormality in LDL-cholesterol, HDL-cholesterol and triglyceride levels [38]. Abnormalities in lipid levels are believed to be key players in the development of coronary heart disease and may be present long before other risk factors occur [38]. Also, the co-existence of hypertension and dyslipidemia is often observed in clinical practice [39] and the risk of cardiovascular events in individuals with concomitant hypertension and dyslipidemia is increased [38, 40]. A recent study demonstrated that low HDL-C levels are associated with an increased risk of hypertension [41]. These findings were similar to observations made in a study conducted in the United States of America, where low levels of HDL-C among the elderly were associated with hypertension and an increased risk of cardiovascular disease [42]. To date, prospective studies that have demonstrated the relationship between controlled blood pressure and plasma lipid levels, particularly HDL-C, are lacking. Therefore, the finding of the association between low HDL-C and uncontrolled hypertension in the present study requires further investigations.

Anti-hypertensive drugs are prescribed mainly to reduce blood pressure and complications associated with the disease. Studies have shown that following clinical guidelines in practice improves the treatment outcomes [43]. Additionally, clinical guidelines, including the South African hypertension guideline, recommend the use of multiple drugs to effectively control blood pressure and reduce the possibility of hypertension-related complications [24]. It is important to note that all the participants in this study had been on anti-hypertensive therapy for at least a year before the commencement of the study. The high prevalence of uncontrolled hypertension occurred, irrespective of the number of drugs and combinations administered. A plausible explanation could be non-adherence to treatment among the study participants. However, the level of adherence to treatment was not explored in the current study. Irrespective of the findings in this study, it is important for clinicians to follow evidence-based guidelines in prescribing anti-hypertensive drugs in order to improve treatment outcomes in these patients.

### Limitations

This study has provided very important insights into the treatment outcome (blood pressure) of individuals attending health facilities for hypertension in the region, but the limitations of the study cannot be ignored. Causal association of the determinants cannot be ascertained, given the cross-sectional design method adopted in the study. Also, the lifestyle assessment depended largely on self-report measures which can be subject to social desirability bias. In addition, the urinary sodium excretion level could have added more objectivity about the consumption of salts among the study participants.

## Conclusion

We found a high prevalence of uncontrolled hypertension in the study setting, possibly attributed to obesity, physical activity and dyslipidaemia. All these determinants should be addressed decisively through collaborative efforts of individuals, clinicians and health authorities. Such collaboration would facilitate achieving the treatment blood pressure targets in the study population. To the best of our knowledge, this study is the first to report the prevalence of uncontrolled hypertension with an association between it and low HDL-C, inadequate physical activity plus obesity among residents of Mkhondo municipality. Furthermore, this study has highlighted the need to implement more aggressive health strategies targeting the identified determinants as well as blood pressure of the patients in order to improve the overall outcomes of care in the rural communities of Mkhondo municipality of Mpumalanga province.

## Supplementary information

**Additional file 1.** Mpumalanga Data Collection Sheet

## Data Availability

All the study materials and data are available from the corresponding author, upon reasonable request.

## Abbreviations

DM: Diabetes mellitus
HDL-C: High density lipoprotein cholesterol
LDL-C: Low density lipoprotein cholesterol
LCDs: Non-communicable diseases
NHLS: National health laboratory services
SEMDSA: Society of endocrinology, metabolism and diabetes of South Africa
TC: Total cholesterol
TG: Triglyceride
