# Supplementary material for: Cross-sectional study of prevalence and determinants of uncontrolled hypertension among South African adult residents of Mkhondo municipality
Source: BMC Public Health. 2020 Jul 6;20:1069. doi: 10.1186/s12889-020-09174-7 (PMC7339580; doi:10.1186/s12889-020-09174-7)
Supplement: Supplementary file 1 — Additional file 1. Mpumalanga Data Collection Sheet [file 12889_2020_9174_MOESM1_ESM.pdf]

Mpumalanga cardiometabolic study

IDENTIFICATION	FORM NUMBER	
	HOSPITAL NUMBER	

1. DEMOGRAPHY		
GENDER MALE = 1 FEMALE = 2		
AGE IN YEARS		
MARITAL STATUS MARRIED =1 SINGLE=2		
ADDRESS	URBAN=1 RURAL=2	

2. SOCIO-ECONOMIC PROFILE	
EDUCATIONAL LEVEL ILLITRATE=1 GRADE 1-6 = 2 GRADE 7- 12 = 3 TERTIARY = 4	
EMPLOYMENT STATUS UNEMPLOYED = 1 EMPLOYED = 2	
MONTHLY INCOME	R

3. ANTHROPOMETRIC MEASUREMENTS			
WEIGHT (KG)		HEIGHT (M)	
BODY MASS INDEX			

CODE:

4. MEDICAL HISTORY

DURATION OF DIABETES (IN YEARS)		
CORONARY ARTERY DISEASE	YES =1 NO = 2	
HEART FAILURE	YES = 1 NO = 2	
HYPERTENSION	YES = 1 NO = 2	
STROKE	YES = 1 NO = 2	
DEPRESSION	YES = 1 NO = 2	
RENAL FAILURE	YES = 1 NO = 2	
FOOT PROBLEMS (DEFORMITIES, ULCERS, AMPUTATIONS)	YES = 1 NO = 2	
EPILEPSY	YES = 1 NO = 1	
OTHER CO-MORBIDITIES:		

5. LIFESTYLE – SMOKING AND ALCOHOL HISTORY

A.

CURRENT SMOKER	1
FORMER SMOKER	2
NEVER SMOKED	3

B.

CURRENT DRINKER	1
QUIT DRINKING	2
NEVER DRANK ALCOHOL	3

6. LEVEL OF PHYSICAL ACTIVITY

PRIVATE CAR	YES = 1	NO = 2
PUBLIC TRANSPORT	YES = 1	NO = 2
WALKING	YES= 1	NO = 2
HOURS SPENT ON TELEVISION	YES = 1	NO = 2
GARDENING	YES =1	NO = 2

7. DIABETIC FOOT CARE

SELF INSPECTION OF THE FEET	YES = 1	NO = 2
APPROPRIATE FOOT WEAR	YES = 1	NO = 2
WALKING BARE FOOTED	YES = 1	NO = 2

8. CURRENT MEDICATIONS

INSULIN	YES = 1	NO = 2	
METFORMIN	YES = 1	NO = 2	
GLIBENCLAMIDE	YES = 1	NO = 2	
GLICLAZIDE	YES =1	NO = 2	
INSULIN + METFORMIN	YES = 1	NO = 2	
INSULIN + GLIBENCLAMIDE	YES = 1	NO = 2	
INSULIN + GLICLAZIDE	YES = 1	NO = 2	
METFORMIN + GLIBENCLAMIDE	YES = 1	NO = 2	
METFORMIN + GLICLAZIDE	YES = 1	NO = 2	
INSULIN + METFORMIN + GLIBENCLAMIDE	YES = 1	NO = 2	
INSULIN + METFORMIN + GLICLAZIDE	YES = 1	NO = 2	
ANTI – HYPERTENSIVE MEDICATIONS			
ASPIRIN	YES = 1	NO = 2	
SIMVASTATIN	YES = 1	NO = 2	

9. SELF-CARE BEHAVIOUR

HOME GLUCOSE MONITORING	YES = 1	NO = 2	
ADHERENCE TO MEDICATION	YES = 1	NO = 2	
ADHERENCE TO DIET	YES = 1	NO = 2	
ADHERENCE TO EXERCISES	YES = 1	NO = 2	
ADHERENCE TO CLINIC APPOINTMENT	YES = 1	NO = 2	

10. PREVIOUS HOSPITALIZATIONS

PREVIOUS EPISODES OF HYPOGLYCAEMIA	
PREVIOUS EPISODES OF HYPERGLYCAEMIA	

11. DIETARY COUNSELLING/ACCESS TO DIETICIAN	YES = 1	NO = 2	

12. SOURCES OF DIABETES INFORMATION

TELEVISION	YES = 1	NO = 2	
HEALTHCARE WORKERS	YES = 1	NO = 2	
LEAFLETS/ LITERATURES	YES = 1	NO = 2	
FAMILY MEMBERS	YES = 1	NO = 2	
FRIENDS AND SUNDRY SOURCES	YES = 1	NO = 2	

13. BLOOD RESULTS

1. HbA1C (MOST RECENT RESULT)

2. RANDOM BLOOD SUGAR
   (LAST THREE VISIT)

3. MEAN OF RANDOM BLOOD SUGAR

4. LIPID PROFILE (MOST RECENT) :
   TOTAL CHOLESTEROL
   HDL – CHOLESTEROL
   LDL- CHOLESTEROL
   TRIGLYCERIDES

5. CREATININE LEVEL

6. CREATININE CLEARANCE

14. HAEMOGLOBIN

15. BLOOD PRESSURE (LAST THREE VISITS):

SYSTOLIC BLOOD PRESSURES (mmHg)

DIASTOLIC BLOOD PRESSURE

MEAN OF THE THREE BPs

16. Additional Information

Salt intake

Low-Moderate ☐

Increased intake ☐

Fruit and Vegetable intake

Never ☐

1-3 time/week ☐

Oil used in food preparation

Vegetable oil ☐

Animal Fat ☐

Never ☐

1-3 times/week ☐

Religious Affiliation

African Traditional ☐

Christianity ☐

Other ☐

How often do you engage in physical activity

Never ☐

1-3 times/week ☐
